# Opposite Effects of Mepyramine on JNJ 7777120-Induced Amelioration of Experimentally Induced Asthma in Mice in Sensitization and Provocation

**DOI:** 10.1371/journal.pone.0030285

**Published:** 2012-01-17

**Authors:** Silke Beermann, Silke Glage, Danny Jonigk, Roland Seifert, Detlef Neumann

**Affiliations:** 1 Institute of Pharmacology, Hannover Medical School, Hannover, Germany; 2 Institute for Laboratory Animal Science, Hannover Medical School, Hannover, Germany; 3 Institute of Pathology, Hannover Medical School, Hannover, Germany; French National Centre for Scientific Research, France

## Abstract

**Background:**

Histamine is detected in high concentrations in the airways during an allergic asthma response. In a murine model of allergic asthma, JNJ 7777120, an antagonist at the histamine H_4_ receptor, reduces asthmatic symptoms, while the histamine H_1_ receptor-selective antagonist mepyramine is virtually without effect. In the present study, we analyzed the effect of combined antagonism at the histamine H_1_ and H_4_ receptors in a murine asthma model in relation to the timing of their application, *i.e.* sensitization or provocation.

**Methodology/Principal Findings:**

Asthma was induced in mice by sensitization and provocation with ovalbumin. JNJ 7777120 and/or mepyramine were injected subcutaneously either during sensitization or during provocation, and typical asthma parameters were analyzed. JNJ 7777120, but not mepyramine, reduced serum concentrations of anti-OVA IgE, inflammatory infiltrations in lung tissue, and eosinophilia in bronchoalveolar-lavage (BAL)-fluids independently of the timing of application. Upon application of JNJ 7777120 plus mepyramine in combination during provocation, mepyramine inhibited the effects of JNJ 7777120. In contrast, when applied during sensitization, mepyramine enhanced the disease-ameliorating effects of JNJ 7777120.

**Conclusions/Significance:**

Our study indicates that both histamine H_1_ and H_4_ receptors play important roles in the course of murine experimental asthma. Unexpectedly, the contribution of these receptors to the pathogenesis differs between the two phases, sensitization or provocation. Since in human asthma, repeated contact to the allergen is not only provocation but also a boost of sensitization, a combined pharmacological targeting of histamine H_1_ and H_4_ receptors could be taken into consideration as an option for the prevention of asthma and maybe other allergic diseases.

## Introduction

Bronchial asthma is a complex disease of the airways, elicited *e.g.* by a type I allergic response, with an increasing incidence worldwide [1]. It is characterized by persistent airway inflammation and hyper-reactivity due to aberrant contractions of smooth muscle cells and mucus production by goblet cells. A widely accepted model of acute airway inflammation is the murine model of ovalbumin (OVA)-induced allergic asthma [2], [3]. In this model, the pathogenesis of asthma can be clearly divided into sensitization and provocation phase. In the sensitization phase, administration of the allergen OVA elicits a Th2-type immune response resulting in the production and systemic distribution of allergen-specific immunoglobulin, of which a substantial proportion is of the IgE isotype. Provocation by repeated inhalation of OVA then induces an acute allergic reaction in the lung leading to local inflammation and airway hyper-reactivity.

An important mediator in type I allergic reactions is the biogenic amine histamine. Histamine concentrations in affected tissue correlate well with severity of the allergic disease [4] and topically applied histamine causes typical allergic symptoms [5]. Histamine exerts its effects through specific receptors on the respective target cells. So far, four histamine receptors have been identified. They belong to the family of G-protein-coupled 7-transmembrane receptors and are referred to as histamine-1 receptor (H_1_R), H_2_R, H_3_R, and H_4_R [6]–[8]. In humans, type I allergic symptoms, such as rhinitis and conjunctivitis, can be controlled effectively by drugs antagonizing the activation of H_1_R, with the exception of bronchial asthma [9]. In mice, genetic deletion of the histamine-forming enzyme L-histidine decarboxylase [10]–[12] or of H_1_R [13], [14] provides beneficial effects in experimental asthma. These data clearly reveal that histamine and presumably also H_1_R are involved in the pathogenesis of bronchial asthma, at least in the murine model. The recently identified H_4_R [15]–[17] is a candidate receptor likely conveying histamine effects in bronchial asthma. Although published data that demonstrate a direct involvement of H_4_R in human asthma are not yet available, in the experimental murine model, asthma symptoms are ameliorated by treating the animals with a H_4_R-antagonist and are reduced in H_4_R^−/−^ mice [18], [19].

In the present study, we asked the question whether H_1_R- and H_4_R-selective antagonists cooperate in the murine model of bronchial asthma, with respect to the two phases of the asthma pathogenesis, sensitization and provocation. The H_1_R-selective antagonist mepyramine [20] and the hH_4_R-selective antagonist JNJ 7777120 [21], [22] have been used for treatment in murine OVA-induced asthma. We show that the ligands in combination cooperatively reduce the allergic reaction when applied during sensitization, whereas, in the provocation phase, mepyramine antagonizes the beneficial effects of JNJ 7777120.

## Results

### 1. JNJ 7777120-induced reduction of asthmatic infiltrations is affected by mepyramine co-administration

**Figure 1:**
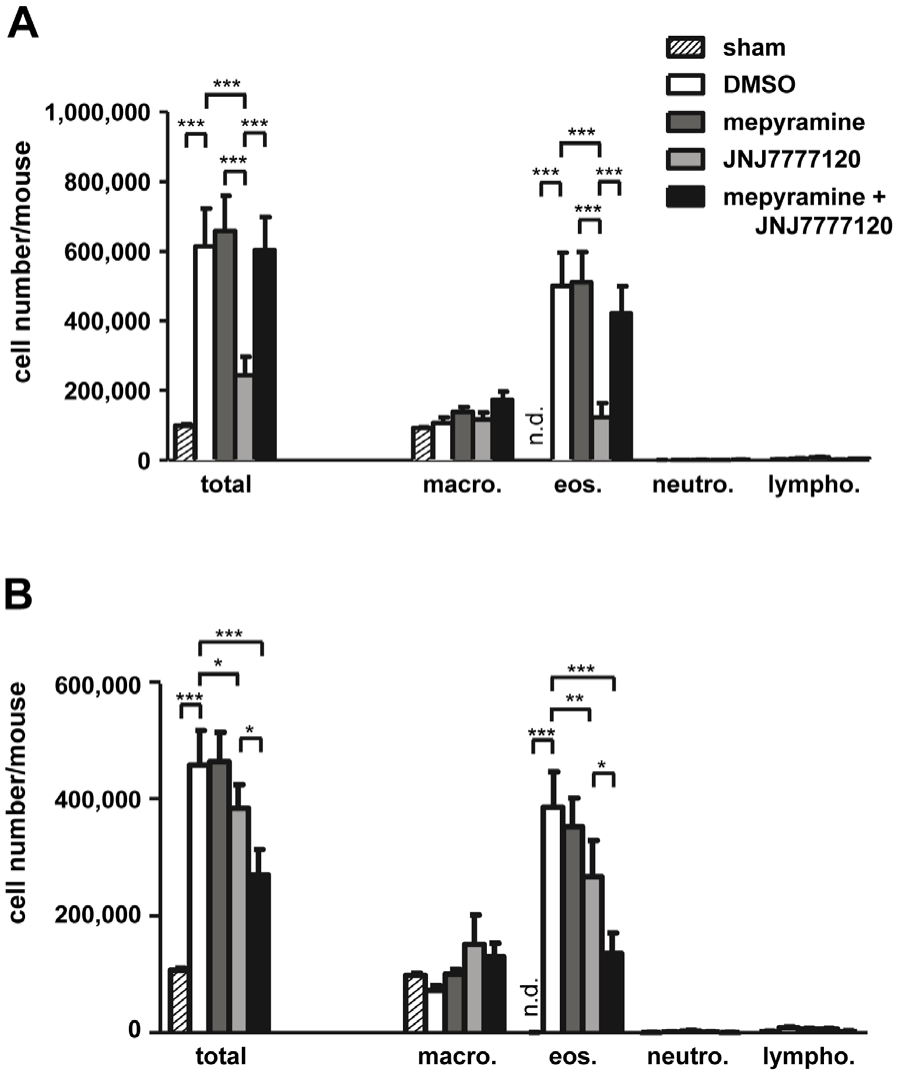
Effect of mepyramine and JNJ 7777120 on BAL-fluid cellularity. Mice were sensitized to, and provoked with, OVA and treated with DMSO, mepyramine, JNJ 7777120, or mepyramine plus JNJ 7777120 either 30 min before each provocation cycle (*A*) or 30 min before and 2 h after each injection for sensitization (*B*). As control, mice were sensitized with PBS (sham), provoked with OVA, and treated with DMSO. Cells in BAL-fluids, collected 24 h after the final challenge, were differentially counted. Data shown are means ± SD (n_(sham)_ = 3, n_(DMSO)_ = 7, n_(mepyramine, JNJ 7777120, or mepyramine+JNJ 7777120)_ = 8; *, p≤0.05; **, p≤0.01; ***, p≤0.005).

In bronchoalveolar lavage (BAL)-fluids of mice with experimental allergic asthma, enhanced numbers of cells are found as compared to those found in sham-sensitized and provoked control mice. This enhanced cellularity of the BAL-fluids is mainly due to the occurrence of high numbers of eosinophils, which are virtually absent in the controls [23]. A comparable enhanced cellularity was observed in asthmatic mice after treatment with the solvent DMSO as well as after treatment with the H_1_R-antagonist mepyramine (Fig. 1). In contrast, treatment of asthmatic mice with the hH_4_R-anatgonist JNJ 7777120 led to a reduction of BAL-fluid eosinophil numbers. These observations were made irrespective of the timing of the treatments, *i.e.* during provocation or during sensitization. Quantitatively, JNJ 7777120 exhibited a more pronounced effect upon its application during provocation (−75% vs DMSO) as compared to its application during sensitization (−31% vs DMSO). A major difference due to the timing of application was observed when the two antagonists, mepyramine and JNJ 7777120, were applied in combination. Upon application during provocation, mepyramine inhibited the effect of JNJ 7777120 on BAL-fluid eosinophil numbers (Fig. 1A). When applied during sensitization, however, mepyramine potentiated the JNJ 7777120 effect, leading to a more pronounced reduction of eosinophil numbers as compared to the treatment with JNJ 7777120 alone (Fig. 1B).

**Figure 2:**
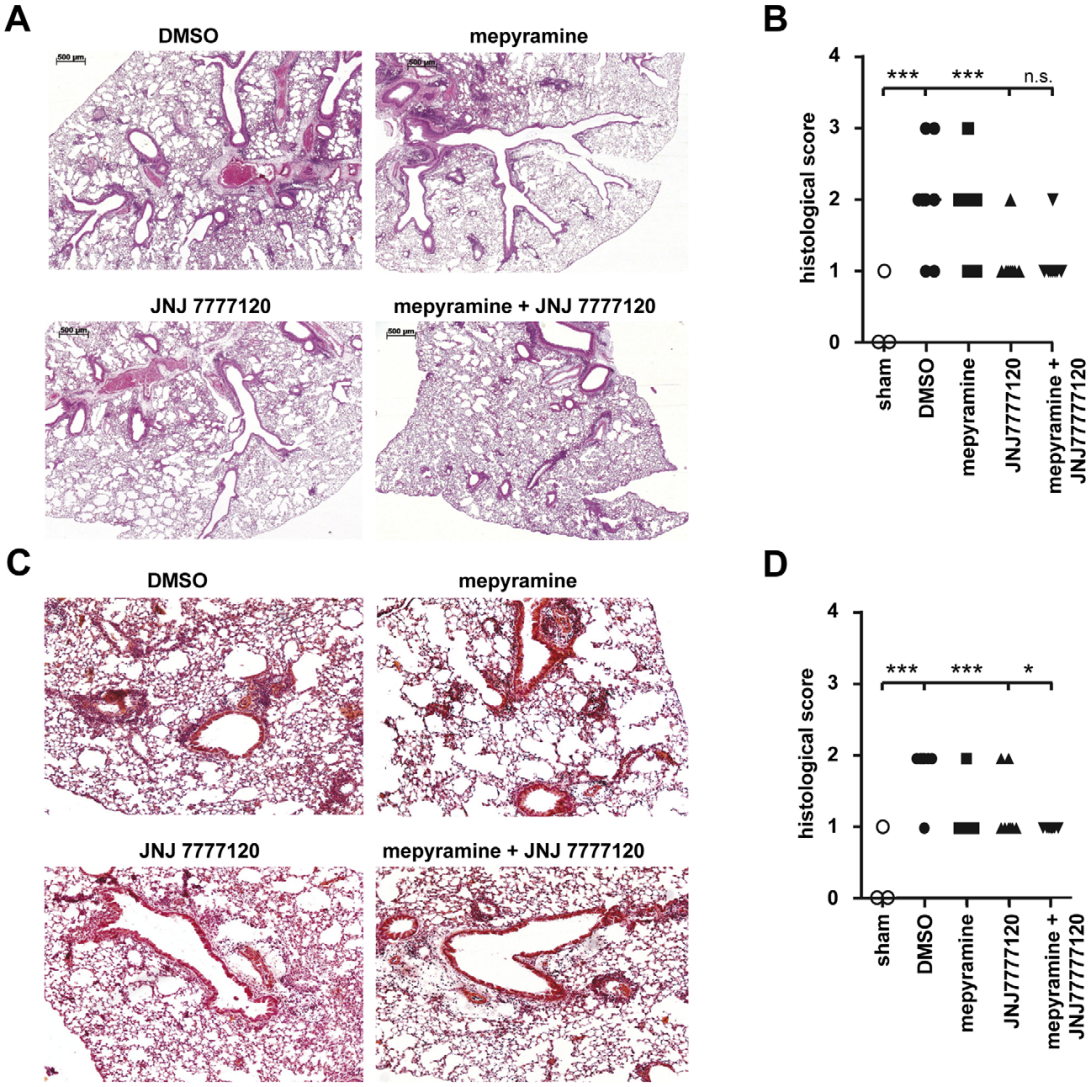
Effect of mepyramine and JNJ 7777120 on airway inflammation. Mice were sensitized to, and provoked with, OVA and treated with DMSO, mepyramine, JNJ 7777120, or mepyramine plus JNJ 7777120 either 30 min before each provocation cycle (*A, B*) or 30 min before and 2 h after each injection for sensitization (*C, D*). *A, C:* Lungs were harvested, processed for histology, and sections were stained with H&E. Representative pictures are shown. *B, D:* Sections as represented in *A, C* were evaluated in a blinded fashion using an index scoring the grade of inflammation. (n_(sham)_ = 3, n_(DMSO)_ = 7, n_(mepyramine, JNJ 7777120, or mepyramine+JNJ 7777120)_ = 8; ***, p≤0.005).

In lung tissue of asthmatic mice, inflammatory infiltrations with lymphoid, monocytic and eosinophilic cells are found around bronchi and vessels [24]. The quantification of the histological sections by a scoring index revealed large inter-individual variability within a treatment group (Figs. 2 B and D). However, in lungs of DMSO-treated asthmatic mice a substantial number of infiltrating cells was found (Figs. 2 A and C). This infiltration was not affected by mepyramine treatment, when performed during provocation (Fig. 2B), but a reduction was found, when mepyramine was applied during sensitization (Fig. 2D). JNJ 7777120 treatment significantly reduced inflammatory lung infiltration upon both, application during provocation and sensitization. Also in both cases, co-administration of mepyramine along with JNJ 7777120 did not reduce the JNJ 7777120 effect; it was rather enhanced when mepyramine and JNJ 7777120 were applied in combination during sensitization. The histology data obtained from mice treated during provocation with the combination of mepyramine and JNJ 7777120 are contradictory to those data obtained by the analysis of BAL-fluids. This discrepancy, however, can be explained methodologically. The grading scheme used to quantify the histologic sections does not differentiate between leukocyte accumulation in the perivenular *vs.* the peribronchial compartments. Thus, it does not reflect the accessibility of individual inflammatory cells by BAL: leukocytes in the bronchi/bronchioles are typically overrepresented in BAL-fluids, while perivenular inflammatory changes are sampled only to a minor degree [25], [26]. This likely accounts for the discrepancy between lung histology data and BAL-fluid data from mice treated with the combination during the provocation phase: eosinophils are concentrated around the veins and venules and not around bronchi and bronchioles.

### 2. The Th2-type immune response in vivo is modulated by mepyramine and JNJ 7777120

**Figure 3:**
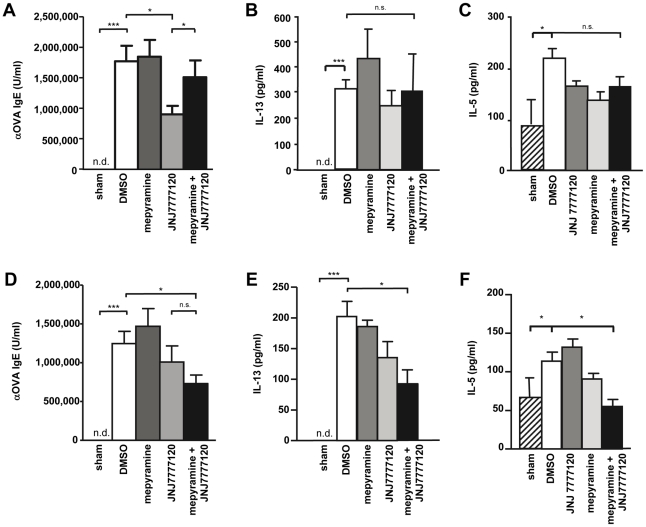
Effect of mepyramine and JNJ 7777120 on Th2 cytokine and anti-OVA IgE concentrations in sera. Mice were sensitized to, and provoked with, OVA and treated with DMSO, mepyramine, JNJ 7777120, or mepyramine plus JNJ 7777120 either 30 min before each provocation cycle (*A, B*) or 30 min before and 2 h after each injection for sensitization (*C, D*). In sera, collected 24 h after the final provocation, IL-13 concentrations were measured by FlowCytomix (*A, C*) and anti-OVA IgE titers were determined by ELISA (*B, D*). Data shown are means ± SD (n_(sham)_ = 3, n_(DMSO)_ = 7, n_(mepyramine, JNJ 7777120, or mepyramine+JNJ 7777120)_ = 8; *, p≤0.05; **, p≤0.01; ***, p≤0.005).

The occurrence of allergen-specific IgE is a hallmark of type I allergic immune responses [27]. Thus, in sera of DMSO-treated asthmatic mice anti-OVA IgE was found, while in sham-sensitized and OVA-provoked mice such antibodies were not detectable (Fig. 3 A and D). Mepyramine treatment of asthmatic mice, either during provocation or during sensitization, did not affect anti-OVA IgE concentrations in sera as compared to DMSO-treated asthmatic mice. In contrast, JNJ 7777120 upon application during provocation reduced the anti-OVA IgE concentration (Fig. 3A), while upon application during sensitization there was only a reduction without statistical significance (Fig. 3D). As already observed by analyzing the cell numbers in BAL-fluids, co-administration of mepyramine along with JNJ 7777120 reversed the JNJ 7777120 effect when applied during provocation (Fig. 3A), but seemed to enhance it when applied during sensitization (Fig. 3D), although this is not supported by the statistical analysis. Nonetheless, upon application during sensitization mepyramine coadminisration did not antagonize the effect of JNJ 7777120, as observed when applied during provocation. Allergic asthma is a Th2-type immune response. Thus, cytokines such as IL- 5 and IL-13 are found in enhanced quantities during such reaction [28]. Consequently, we found substantial concentrations of IL-5 and IL-13 in sera of asthmatic mice, while they were lower or undetectable in sera of sham-sensitized and OVA-provoked mice (Fig. 3 B, C, E, F). In this assay, key cytokines typical for other types of Th cell responses, such as IL-17 and IFNγ were either undetectable or unaltered, respectively (not shown). The application of mepyramine or JNJ 7777120, either alone or in combination, during provocation did not affect the serum concentration of IL-13 (Fig. 3 B) or IL-5 (Fig. 3 C). In contrast, upon application during sensitization, JNJ 7777120 in combination with mepyramine significantly reduced the IL-13 and IL-5 serum concentrations in comparison to the DMSO control, while both ligands alone were without significant effect (Fig. 3 E, F).

### 3. The T cell response in vitro is modulated by mepyramine and JNJ 7777120

**Figure 4:**
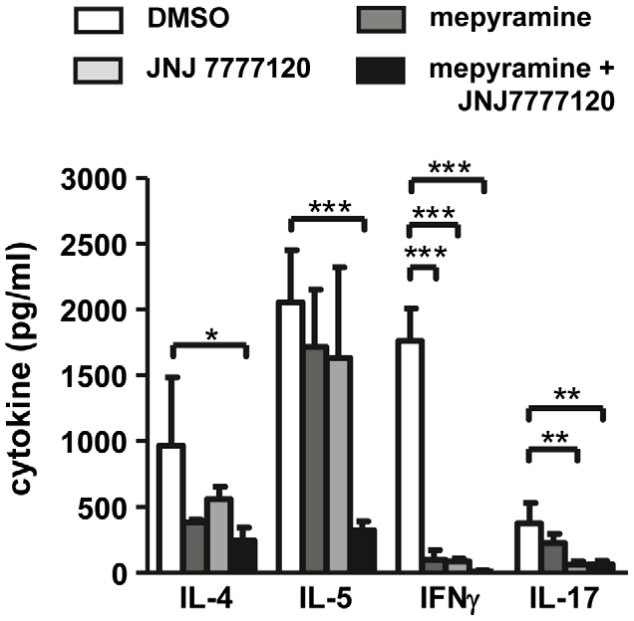
Effect of mepyramine and JNJ 7777120 on the T cell cytokine production of lymph node cells after in vitro re-stimulation. Mice were sensitized to OVA and treated with DMSO, mepyramine, JNJ 7777120, or mepyramine plus JNJ 7777120 30 min before and 2 h after each injection for sensitization. 48 h after the last injection, single cells suspension out of mesenteric lymph nodes were re-stimulated *in vitro* with OVA for 48 hours. Thereafter, accumulated cytokines in the supernatants were quantified by a multiplex assay. Data shown are means ± SD (n_(each group)_ = 3*, p≤0.05; **, p≤0.01; ***, p≤0.005).

Cells obtained from mesenteric lymph nodes of mice treated during sensitization with DMSO, mepyramine, JNJ 7777120, or mepyramine plus JNJ 7777120, which contained comparable amounts of CD4^+^ and CD8^+^ cells (not shown), were re-stimulated *in vitro* with OVA and analyzed for the expression of T cell specific cytokines. In cells from DMSO-treated mice, the Th2-type cytokines IL-4 and IL-5 were substantially expressed as well as IFNγ and IL-17, which are prototypical for Th1 and Th17 cells, respectively. These cytokines were reduced in supernatants of cells obtained from mice treated with mepyramine plus JNJ 7777120 (Fig. 4). The treatment of the mice with mepyramine or with JNJ 7777120 alone led to the reduced *in vitro* synthesis of IFNγ or of IFNγ and IL-17, respectively, while IL-4 and IL-5 were not altered significantly (Fig. 4). Other cytokine, IL-1α IL-2, IL-6, IL-10, were detected in the supernatants, too, but without alterations of their abundance due to the mepyramine/JNJ 7777120 treatment of the mice (not shown).

## Discussion

In the present study we analyzed the effects of mepyramine and JNJ 7777120, antagonists at the H_1_R and H_4_R, respectively, in a murine model of experimentally induced asthma. Particularly, we compared their effects upon application during the sensitization and the provocation phases.

While treatment of asthmatic mice with mepyramine had virtually no effect on the disease parameters analyzed, asthmatic symptoms were reduced by the application of JNJ 7777120. These effects of the single treatment were independent of the timing of application, sensitization or provocation. Similar observations with JNJ 7777120 have already been published elsewhere [19], while it has been shown previously, that mepyramine at 20 mg/kg body weight during provocation was able to reduce BAL-fluid eosinophilia [29]. This discrepancy is most likely due to differences in the mode of application, since in the cited study, mepyramine was injected twice daily and, in addition, already the day before the initiation of the provocation phase [29].

The major topic of the present study is the application of the H_4_R antagonist JNJ 7777120 [21] in combination with the H_1_R antagonist mepyramine [20], either during the sensitization phase or during the provocation phase. We observed an efficient modulation of the JNJ 7777120 effect by mepyramine, which was strictly dependent on the timing of application. Upon application during provocation, mepyramine inhibited the effect of JNJ 7777120, while during sensitization mepyramine enhanced the symptoms-reducing effect of JNJ 7777120 (summarized in table 1). This difference can be explained by the different target cells, which are affected by histamine in the respective phase, sensitization and provocation.

**Table 1:** Comparison of the effects of mepyramine, JNJ 7777120, and mepyramine+JNJ 7777120 on parameters of murine OVA-induced experimental asthma.

application timing	provocation			sensitization		
treatment	mepy.	JNJ 7777	mepy. +JNJ 7777	mepy.	JNJ 7777	mepy. +JNJ 7777
serum αOVA IgE	**→**	**↓**	**→**	**→**	**(↓)**	**↓**
serum IL-13	**→**	**(↓)**	**→**	**→**	**(↓)**	**↓**
lung histology	**→**	**↓**	**↓**	**↓**	**↓**	**↓**
BALF eosinophilia	**→**	**↓**	**→**	**→**	**↓**	**↓↓**
*impact of mepy. on JNJ 7777 treatment*	***antagonistic***			***synergistic***		

The effects of mepyramine, JNJ 7777120, or mepyramine plus JNJ 7777120 applied during either the provocation phase or the sensitization phases on selective parameters observed in the experimental asthma model in this study were compared to the treatment with the solvent DMSO in the respective phase, and evaluated as not altered [**→**], slightly reduced [(**↓**)], reduced [**↓**], and strongly reduced [**↓↓**]. (mepy.: mepyramine, JNJ7777: JNJ 7777120).

Sensitization basically is an immunoreaction against the allergen, which takes place in the lymph nodes. Here, histamine is produced most likely by dendritic cells and T cells and acts in an autocrine manner, affecting T cells polarization [8]. In dendritic cells and T cells, the H_1_R is involved in the regulation of cytokine production [30], [31] and the H_4_R regulates cytokine production, antigen presentation, and cell migration [13], [19], [32]. Thus, antagonism at both, H_1_R and H_4_R, may reduce the activation of dendritic cells and/or T cells. However, so far there is no proof, that H_1_R and H_4_R are expressed in parallel on the same cell *in vivo*, since highly specific antibodies to detect these receptor proteins in native cells are not available [33]. Therefore, we cannot exclude the possibility, that mepyramine and JNJ 7777120 act on different target cells (two dendritic cell subsets, two T cell subsets, dendritic cell and T cell), resulting in the observed effect. Moreover, additional mechanism, constrained to the *in vivo* situation, seem to be involved in this process, since the synergistic effect of the mepyramine plus JNJ 7777120 treatment was not detected in the *in vitro* assay using lymph node cells obtained from treated mice.

During provocation, histamine is released by mast cell- or basophil-degranulation resulting in rather high local concentrations and affects peripheral cells such as mast cells, endothelial cells, eosinophils, or polarized T cells. *E.g.* products released by Th2 cells regulate growth and activation of eosinophils, thus, playing a critical role in the pathogenesis of acute and chronic asthma [34]. Here, the H_4_R may enhance the activation of effector cells, while the H_1_R, either directly or indirectly via other cells, reduces their activation.

Alternatively, the different effects due to the timing of application of mepyramine and JNJ 7777120 may also be a consequence of different histamine concentrations in the active tissues [33], which are rather low in lymph nodes in contrast to acute asthmatic lungs [4]. Since JNJ 7777120 behaves as a partial agonist at the recombinant murine H_4_R expressed in Sf9 cells [22], [35], its actual function *in vivo* may depend on the local ratio of histamine and JNJ 7777120, and thus may differ between the two tissues [36]. Moreover, we do not know the expression levels of the two receptors. When expressed on the same cell, the ratio of their expression levels may differ between cells, resulting in different effects as observed upon application of meypramine plus JNJ 7777120 in the provocation or the sensitization phase. In this scenario also the concept of functional selectivity may apply [37]–[39]. Particularly, in the lymph node JNJ 7777120 may act by inhibition of H_4_R-mediated G-protein signaling [40], which is potentiated by mepyramine, while in the periphery JNJ 7777120 may act via activation of ß-arrestin [41], which is inhibited by mepyramine.

In conclusion, in the present study we provide evidence that histamine via both, the H_1_R and the H_4_R, plays a critical role in the pathogenesis of murine asthma. The contribution of the two histamine receptors, however, differs between the two phases of the pathogenesis, *i.e.* sensitization and provocation. This result is unexpected and cannot be sufficiently explained by the current paradigm of histamine receptor function. Therefore, the cellular and molecular bases for this difference have yet to be identified, inasmuch as the involvement of the H_1_R and the H_4_R in human asthma has not been analyzed so far. However, our data indicate a potential role for histamine receptor-antagonists also in the therapy of human asthma.

The present study is limited by to the number of parameters analyzed in order to characterize the type of immune response. In order to unambiguously demonstrate the bias towards a Th2-type response and/or its reversion towards a Th1- or a Th17-type response, more markers need to be addressed. However, the major topic of the present study is not a detailed analysis of the type of T cell response, but the comparison of the effects of mepyramine plus JNJ 7777120 as functions of time of their application, sensitization or provocation. Although our data basically support our conclusions, from a therapeutical point of view, functional analysis such as lung function measurements are clearly missing in this study. These technically challenging experiments will be provided in a follow-up study. Moreover, additional parameters characterizing in a detailed manner the underlying immune response will have to be determined in future studies. Lastly, the pharmacokinetics of the applied antagonists, mepryamine and JNJ 7777120, may differ from each other, possibly resulting in the observed differences.

## Materials and Methods

### 1. Ethics statement

All animal work has been conducted according to relevant national and international guidelines and was performed under a protocol approved by the local governmental authority (Niedersächsisches Landesamt für Verbraucherschutz und Lebensmittelsicherheit; approval ID 33.9-42502-04-08/1550).

### 2. Mice

Female Balb/c mice of 8 weeks of age were obtained from Elevage Janvier (Le Genest-Saint-Isle, France), and housed in community cages on a 12 h light cycle and fed mouse chow and water ad libitum in the animal facility of the Hannover Medical School. In order to enable their acclimatization, mice were kept for at least 2-weeks in the animal facility before starting experimental applications.

### 3. Sensitization and allergen challenge of mice

Experimental asthma was induced by sensitization with endotoxin-free ovalbumin (OVA; Hyglos, Bernried, Germany) and provocation with OVA grade V (Sigma-Aldrich, Taufkirchen, Germany). The use of these two different preparations of OVA has been shown in preliminary experiments to result in an optimal asthmatic response. For sensitization, the mice were injected intraperitoneally (i.p.) on days 1 and 14 with 10 µg of OVA, which was absorbed to 1.5 mg of PBS-suspended aluminium hydroxide (alum, Pierce Biotechnology, Rockford, IL, USA). In control experiments, mice were sham-sensitized with alum/PBS. Provocation was carried out at days 21–24 by daily exposition of mice to 1% (m/v) OVA/PBS for 20 min in a custom made nebulizer box in which the mice could move freely.

### 4. H_1_R and H_4_R antagonist administration

The H_1_R-selective antagonist mepyramine (Sigma-Aldrich) and the H_4_R selective antagonist JNJ 7777120 (1-[(5-chloro-1*H*-indol-2-yl) carbonyl]-4-methylperazine, kindly provided by Dr. Armin Buschauer, University of Regensburg, Germany) were dissolved in 20% (v/v) DMSO to obtain solutions of 10 mM. They were administered in final doses of 20 mg/kg body weight [19], [29]. The mice sensitized and provoked with OVA were divided into four experimental groups, which were treated either with 20% (v/v) DMSO, with only mepyramine, with only JNJ7777120, or with a combination of mepyramine and JNJ777120. Treatments were performed by subcutaneous (s.c.) injections, using a total volume of 100 µl with each application. When applied during the provocation phase, mice were treated 30 min before each OVA exposure. For application in the sensitization phase, DMSO or antagonists were administered 30 min before, and additionally 2 h after, OVA injections. In preliminary experiments, it has been found that the second administration of JNJ 7777120 two hours after OVA injection is necessary to obtain an effect on the asthmatic response [19]. A schematic representation of the experimental groups is outlined in table 2.

**Table 2:** Experimental groups of mice.

*group*	*n*	*sensitization*	*provocation*	treatment
sham	3	PBS	Ova	DMSO
DMSO	7	Ova	Ova	DMSO
mepyramine	8	Ova	Ova	mepyramine
JNJ 7777120	8	Ova	Ova	JNJ 7777120
**mepyramine +** **JNJ 7777120**	8	Ova	Ova	mepyramine +JNJ 7777120

Mice, divided into the five groups indicated on the left, were either sham-sensitized with PBS or sensitized with OVA. Provocation was carried out by application of OVA in a nebulized form. Mice were treated either during sensitization or during provocation with DMSO (sham; DMSO) or with mepyramine and/or JNJ 7777120.

### 5. Animal dissection

Mice were sacrificed with an i.p. injection of a mixture of rompun (70 mg/kg bodyweight; Bayer, Leverkusen, Germany) and ketamine (230 mg/kg bodyweight; Albrecht, Aulendorf, Germany). Mice were bled by heart puncture and sera were prepared and frozen at −80°C. Lungs were individually lavaged in situ with 1.0 ml aliquots of sterile PBS (bronchoalveolar lavage: BAL), and afterwards fixed in 4% (m/v) formaline and embedded in paraffin.

### 6. Differential cell count

Total cell counts in BAL-fluids and viability were determined by trypan blue exclusion using a Neubauer chamber. Leukocyte populations (eosinophils, neutrophils, macrophages or lymphocytes) were counted in cytospins from BAL-fluids stained with Diff-Quik (Medion Diagnostics, Dueningen, Germany). A total of 400 cells were counted in each sample.

### 7. Histology

Fixed and paraffin-embedded lungs were cut into 4 µm slices and stained with hematoxilin/eosin. Representative microphotographs were taken using a Zeiss Axioskop 40 microscope with a Zeiss AxioCam MRc camera. From each lung six sections (containing hilus-structures and peripheral tisuue) of both lungs were evaluated. Quantification of the bronchial inflammation was performed according to a scoring index adapted from [42] ad outlined in table 3.

**Table 3:** Histological scoring index.

grade	• Histological appearance
0	• no focal infiltration or peribronchial or perivascular inflammatory infiltrates
1	• cuffs of leukocytes and eosinophils mostly around central bronchi and veins in a thin layer• more lymphocytes than eosinophils
2	• cuffs of leukocytes and eosinophils around central bronchi and veins in a layer thicker than grade 1• lymphocytes/eosinophils ratio: 50/50• no/some aggregates of inflammatory cells in lung parenchyma
3	• cuffs of leukocytes and eosinophils around central bronchi and veins and up to 2/3 of periphery• lymphocytes/eosinophils ratio: 50/50• some aggregates of inflammatory cells in lung parenchyma
4	• cuffs of leukocytes and eosinophils around all visible bronchi and veins up to pleura• eosinophilic cells diffuse in lung parenchyma• aggregates of inflammatory cells diffuse in lung parenchyma

The scoring index was adapted from Mehlhop et al [42] with minor modifications. According to this index, inflammatory infiltrations observed in lung tissues sections (figure 2) were quantified.

### 8. In vitro stimulation of lymph node cells

Mice were sacrified 48 h after the last injection for sensitization. Single cell suspension were prepared out of mesenteric lymph nodes by teasing the organs and two times of repeated washing in RPMI 1640 medium, supplemented with 5% [v/v] fetal calf serum (FCS), 2 mM L-glutamate, 50 µM 2-mercaptoethanol, 100 U/ml penicillin, and 100 µg/ml streptomycin (all from PAA, Pasching, Austria). Cells were resuspended in RPMI 1640 plus supplements and 5×10^5^ cells/200 µl*well were plated in 96-well plates (Nunc. Roskilde, Danmark) and re-stimulated *in vitro* with 50 µg/ml OVA for 48 hours. Thereafter, cell-free supernatants were harvested and either analyzed immediately or stored at −80°C until use.

### 9. Evaluation of cytokine/chemokine concentrations

The cytokine concentrations in sera or in cell culture supernatants were measured using the Mouse Th1/Th2 kit of the FlowCytomix system (BenderMedSystems, Vienna, Austria). The assay was performed according to the instructions supplied by the manufacturer.

### 10. Mouse anti-OVA IgE ELISA

Serum concentrations of OVA-specific IgE were analyzed by ELISA using a mouse-specific anti-IgE-antibody. Briefly, OVA-coated microtiter plates (Nunc, Roskilde, Denmark) were incubated with test sera, diluted 1∶200 in PBS containing 5% (w/v) BSA, and serial dilutions of a standard serum obtained from a hyper-immunized mouse. Murine IgE was detected by subsequent incubation with a biotinylated rat anti-mouse IgE antibody (BD/Pharmingen, Franklin Lakes, NJ, USA) and peroxidase-coupled streptavidine (Invitrogen-Biosource, Carlsbad, CA, USA). Peroxidase-dependent conversion of TMB was quantified by reading at 405 nm. Titers were calculated according to the standard serum obtained from a hyper-immunized mouse, which was set arbitrarily at 1×10^6^ U/ml.

### 11. Statistical analysis

If not stated otherwise, statistical analyses were performed by calculating means ± SD for each parameter within an experimental group. Analysis of significance was performed using one-way ANOVA with Bonferroni post test for linear parameters or the chi-square (Fisher's exact) test for categorical data (both GraphPad Prism 5). p-Values of <0.05 (*), <0.01 (**), and <0.005 (***) were considered significant.
